# Cluster Headache Management: Evaluating Diagnostic and Treatment Approaches Among Family and Emergency Medicine Physicians

**DOI:** 10.3390/medicina61030437

**Published:** 2025-02-28

**Authors:** Buse Rahime Hasirci Bayir, Ezgi Nazli, Can Ulutas

**Affiliations:** Haydarpaşa Numune Eğitim ve Araştırma Hastanesi, 34668 İstanbul, Turkey; ezgigoger@gmail.com (E.N.); canulutas222@hotmail.com (C.U.)

**Keywords:** cluster headache, family medicine, emergency medicine, misdiagnosis, knowledge

## Abstract

*Background and Objectives*: Cluster headaches (CHs) are one of the most painful primary headaches and negatively affect the lives of patients due to misdiagnosis. Family medicine (FM) and emergency medicine (EM) physicians are one of the most important steps in making the correct diagnosis and directing patients to headache specialists. In this study, the knowledge and management approaches of these two groups regarding CH were evaluated. *Materials and Methods*: Two online questionnaires were developed to gather the demographic data of physicians and to assess their knowledge about the characteristics, diagnosis, and treatment of CHs. *Results*: A total of 120 FM doctors and 98 EM doctors participated in this study. Answers about diagnostic criteria were similar in both groups. It was found that 70% of the participating physicians had concerns about misdiagnosing cluster headaches, and only 15% considered themselves sufficiently knowledgeable on the topic. Additionally, nearly half of the physicians were unaware that autonomic symptoms are mandatory for diagnosis and believed that NSAIDs are effective in treatment. *Conclusions*: In our study, for the first time, EM and FM physicians’ knowledge about the diagnosis and treatment of and professional competence in CHs was evaluated. It was found that the participants had knowledge about CHs but still considered themselves incompetent. For the correct and early diagnosis and for the proper management of CHs, EM and FM physicians, who can be called gatekeepers of CHs, need more medical education-based strategies.

## 1. Introduction

Cluster headaches (CHs) are a primary headache in the group of trigeminal autonomic cephalalgias [1]. According to epidemiologic surveys, the frequency of CHs in the population is 0.1–0.2% [2]. It is more common in men, with a mean ratio of 4.7:1 compared to women [3]. This ratio increases even more in chronic forms (11:1) [4]. However, with the increase in correct diagnoses in women, the gap in prevalence between genders is narrowing [5]. CHs most commonly affect young and middle-aged people between the ages of 20 and 40, and their incidence decreases with age [6]. The presence of CHs in first-degree relatives increases the risk of developing the condition by 5 to 18 times [7,8].

According to the International Classification of Headache Disorders-3 (ICHD-3) criteria, CHs are defined as follows: ‘attacks of severe, strictly unilateral pain, which is orbital, supraorbital, temporal or in any combination of these locations, lasting 15–180 min and occurring from once every other day to eight times a day. The pain is associated with ipsilateral conjunctival injection, lacrimation, nasal congestion, rhinorrhea, forehead, and facial sweating, miosis, ptosis and/or eyelid edema, and/or with restlessness or agitation. In episodic form, pain-free periods lasting at least three months. In chronic form, there is no remission between attacks, or it lasts less than three months’ [9]. CH attacks follow a circadian rhythm, most frequently occurring around 2 a.m., and exhibit seasonal patterns, most commonly between April and October [10,11]. The pathophysiology of cluster headaches is similar to migraine, which is the most commonly misdiagnosed condition [12]. Hypothalamic and trigeminovascular activation plays an important role in the pathogenesis of CHs, similar to migraine [13]. It has been suggested that autonomic findings develop due to parasympathetic hyperactivity, and circadian patterns and behavioral changes develop due to hypothalamic dysfunction [14,15].

The pain in CH attacks is very severe and is thought to be even stronger than the pain of childbirth and kidney stones [16]. Since the severity of pain in CHs can lead to suicidal ideation in patients, they are also referred to as a ‘suicide headache’. Pain attacks affect patients’ daily lives and cause socio-economic losses [17,18]. In a study conducted by Pohl et al., CH patients’ feelings of being understood by colleagues and friends decreased as the number of sick days increased [19]. Since the pain is very severe, and includes throbbing, piercing, or stabbing pain, it can cause anxiety and difficulty in concentration in patients. Depression is 2.8 times more common in patients with CHs than in healthy individuals [20]. In order not to increase the frequency of attacks, patients should pay attention to triggers [21,22]. Alcohol consumption, high altitude, and poor sleep hygiene are the most important triggers [23,24]. Therefore, physicians should warn their patients about triggers such as shift work. Recognizing CHs when the first attacks begin, as well as managing and treating them correctly, is very important to improve the quality of life of patients.

Physicians who first evaluate a CH patient, including neurologists, can sometimes make an incorrect diagnosis, such as migraine, sinusitis, trigeminal neuralgia, or dental disorders. Therefore, unnecessary procedures such as sinus washout or teeth extraction can be performed on patients [25]. The most important reasons for misdiagnosis are clinicians’ inadequate knowledge about and experience with the disease and their inability to describe the patient’s pain correctly [26]. Since genetic factors are known to play an important role in the development of CHs, and since the incidence is higher in patients with a family history of CHs, one patient’s early and correct diagnosis can affect the lives of many people [7,27].

Before consulting a headache specialist, CH patients may consult physicians from many different branches. Family medicine (FM) physicians are the first physician group that patients often consult. They are one of the most important gatekeepers for CHs. In our country, emergency medicine services are predominantly provided by emergency medicine (EM) specialists. General practitioners rarely work in emergency departments. In healthcare institutions where there is no emergency specialist, patients with very severe headaches such as CHs are often referred to the emergency departments of tertiary hospitals where emergency specialists are available in order to exclude secondary causes. Due to very severe attacks, patients are evaluated in emergency services, which are one of the first centers to which patients present. Since the FM and EM groups have the highest probability of first evaluating CH patients, the knowledge of these two groups about CHs was evaluated.

The lack of training of FM and EM specialists who are not specifically trained and experienced in the management of headache disorders leads to CHs being underdiagnosed and often undertreated. The early recognition of CHs and reducing misdiagnosis are closely related to FM and EM physicians’ knowledge level and experience with the disease, as they are likely to assess the patients at the first attack. This study aimed to evaluate the current diagnosis, treatment, and management approaches of FM and EM physicians about CHs, which require early diagnosis and the correct treatment.

## 2. Method

Two detailed questionnaires were designed by the authors to explore the understanding of CHs among FM and EM doctors (Supplementary S1 and S2). The pilot study was first evaluated by a headache specialist who was not part of the team that prepared the questions. After the necessary corrections were made, the survey was conducted on a small group (10 EM and 10 FM physicians) to evaluate its understandability. The survey was finalized based on the feedback received from the participants. The last form of the questionnaire included 29 and 32 questions in total. Questions were planned in open-ended, yes/no, true/false, Likert, and multiple-choice formats. They aimed to collect the demographic data of physicians and find out their knowledge about the characteristics, diagnosis, and treatment of CHs, as well as their approach to the condition.

Between 1 and 31 November 2023, the invitation to participate in the survey was sent to all accessible FM and EM physicians, from residents to specialists at every level, via social media. There was no age restriction. Physicians were asked to participate in the questionnaire about CHs after giving informed consent via the Internet. Each participant was allowed to complete the survey only once. There was no data loss, as all participants completed the survey correctly.

All questions were mandatory. There was no time limit for completing the survey and no specific warning was placed in the questionnaire about not receiving support from the literature. Participants were divided into two groups: the FM group and the EM group. The responses were compared statistically between the two groups to evaluate their approaches to the diagnosis and treatment of CHs, in addition to the participants’ thoughts on their competence in managing CHs.

The study was approved by the Hamidiye Medical Ethics Committee of the University of Health Sciences on 13 October 2023 (approval number: 2023/18). The consent form for participation was placed at the beginning of the survey; an informative statement stated that participants agreed to participate in this study if they completed it. For data analysis, the SPSS version 25 package program was used. Descriptive analyses were performed, and percentage values were given for categorical variables. The chi-square test was used for comparisons of categorical data between FM and EM groups. Confidence interval and effect size were calculated for all *p* values found to be statistically significant. Statistical significance was accepted as *p* < 0.05.

## 3. Results

**Figure 1:**
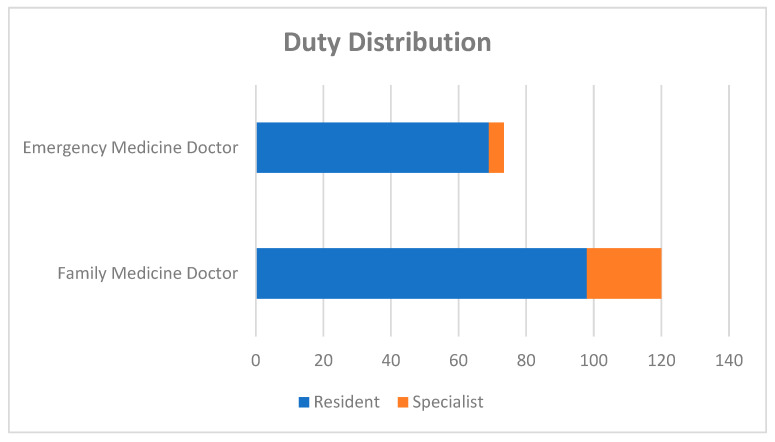
Duty distribution among family medicine and emergency medicine groups.

**Figure 2:**
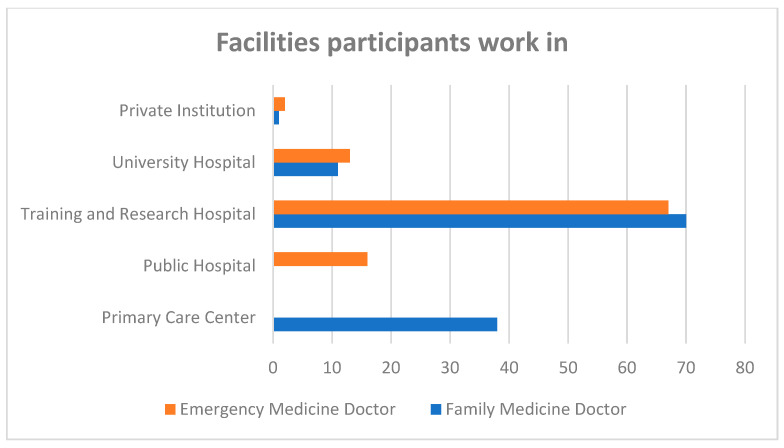
Facilities participants work at among family medicine and emergency medicine groups.

A total of 120 FM doctors and 98 EM physicians participated in this study. The female percentage was 64.2% in the FM group and 53.1% in the EM group. The average age was 32.32 ± 6.73 (min-max: 25–60) in the FM group and 31.07 ± 5.29 (min-max: 24–49) in the EM group. The average duration of professional experience was 6.44 ± 5.51 years (min-max: 1–35) and 4.65 ± 4.75 years (min-max: 1–26), respectively. Most of the participants in both groups were residents (81.7% in the FM group, 70.4% in the EM group, 76.6% in total) (Figure 1). The majority of the participants in both groups worked in training and research hospitals in Turkey (62.84% in total) (Figure 2).

Answers regarding the diagnosis and treatment of CHs were compared in two groups (Table 1). The most preferred and non-statistically significant answers in both groups were as follows: CHs are a primary headache; it is not a migraine subtype; the headache is unilateral and severe; it lasts 15–180 min; it is more common in men; it has a seasonal pattern; oxygen therapy is given in the treatment of attacks; and smoking and alcohol are the most important triggers of CHs. While the fact that autonomic findings are unilateral and accompany CHs during headaches was better known by the FM group (*p*: 0.033), the fact that lacrimation and conjunctival hyperemia are the most frequently seen autonomic findings was known at a similar rate in both groups. Both groups stated that paracetamol and nonsteroidal anti-inflammatory drugs (NSAIDs) were effective in CH treatment. The statement that ‘neuroimaging should be performed on every patient suspected of having CH’ was statistically significant in the FM group (*p*: 0.002). The difference, with a 95% confidence interval (CI) (8.5–33.9%), was statistically significant and the Cramer’s V value of 0.24 indicates a moderate effect size (ES). These findings suggest that FM physicians are more likely than EM physicians to support the universal recommendation of neuroimaging.

For the diagnosis of CHs, the rate of using the classification of the International Headache Society in the FM group was 16.7%, and that in the EM group was 16.3%. In the EM group, 38.8% of participants stated that triptans were effective in treating CHs. In the EM group, 92.9% of participants reported monitoring CH patients in the emergency green zone, while only 7.1% reported monitoring CH patients in the red zone. Participants stated that patients receive attack treatment within an average of 34.07 ± 30.72 min (min-max: 3–180). Additionally, 43.9% of the participants indicated that they administered oxygen therapy with a face mask to every patient suspected of having a CH.

Participants’ thoughts on their professional competence in CHs are evaluated in Table 2. The FM group were more likely to state that they could recognize CH patients easily compared to the EM group (*p*: 0.029, 95% CI 3.3–29.5%, ES by Cramer’s V ≈ 0.18). Misdiagnosis was a greater concern among FM physicians compared to EM physicians (*p*: 0.029, 95% CI 3.2–27.4%, ES by Cramer’s V≈ 0.18). Regarding the statement ‘I have adequate knowledge about CH’, 50.0% of FM physicians disagreed, compared to 28.6% of EM physicians (*p*: 0.006). With an estimated 95% CI (8.7–34.1%) and ES (Cramer’s V ≈ 0.22), it can be stated that FM physicians are more likely to perceive a deficiency in their knowledge about CHs compared to the EM group. The EM group was more likely to believe that CH patients should be referred to the neurology clinic (*p*: 0.008, 95% CI 5.4–24.6%, ES by Cramer’s V ≈ 0.21). A considerably higher proportion of FM physicians felt less confident in their knowledge about treatment options for acute CH attacks than EM physicians (*p*: 0.003, 95% CI 7.9–26.3%, ES by Cramer’s V ≈ 0.225). The FM group also reported that 72.5% refer CH patients with acute attacks to the emergency department.

To summarize our results, a total of 120 FM doctors and 98 EM doctors participated in this study. The age, gender, ratio of assistants to specialists, and the institutions participants worked at were similar in both groups (Figure 1 and Figure 2). While answers about diagnostic criteria and triggers were similar in both groups, the fact that autonomic findings are unilateral and accompany CHs during headaches and the fact that neuroimaging should be performed on every patient suspected of having a CH were better known by the FM group (Table 1). Participants’ thoughts on their qualifications to treat CH were evaluated. The FM group were more likely to state that they could recognize CH patients easily compared to the EM group, but they would be concerned about misdiagnosis, and they did not think their knowledge about CHs was sufficient. The EM group stated that CH patients should be referred to the neurology clinic and that they had sufficient knowledge about attack treatment. The approaches of both groups were similar in that they would feel more confident if they learned more about CHs (Table 2).

## 4. Discussion

The approaches of the FM and EM groups to the diagnosis, treatment, and follow-up processes of CHs were evaluated. It was observed that awareness and knowledge levels regarding CHs were high in both groups. There are many studies in the literature evaluating the experiences of CH patients during the diagnosis and treatment process [28,29]. However, there is only one study with a very limited number of participants (eight neurologists and eight general practitioners) evaluating the experiences of physicians with CHs [26]. In CHs, where the delay in diagnosis can be up to 5 years and is accompanied by very severe attacks, FM and EM physicians are often the CH patient’s first point of call [30]. The correct diagnosis and management of CHs by these two groups will be the most important steps in reducing both misdiagnoses and the burden of disease.

The high rate of correct answers to questions about CH diagnosis, as well as about the location, gender predominance, severity, duration, and accompanying autonomic findings of CHs, indicates that physicians in both the EM and FM groups were well aware of the diagnostic criteria for CHs. These findings are promising for CHs, for which late diagnoses and misdiagnoses are unfortunately common. One study shows that only 21% of patients receive a correct diagnosis when their attacks first begin [30]. Migraine is the most common misdiagnosis, followed by trigeminal neuralgia, dental disorders, and sinusitis. Delays due to misdiagnosis also lead to anxiety, depression, and even suicidal thoughts in patients [31,32]. Unfortunately, the rate of using the ICHD diagnostic criteria for CHs in both groups was a low rate of 16%. Educating physicians on the importance of diagnostic criteria is necessary to reduce misdiagnoses. The Erwin test, which is a fast and easy-to-apply test, can be preferred for a rapid assessment before using diagnostic criteria. The Erwin test has 85% sensitivity and 89% specificity for the diagnosis of CHs. For an accurate diagnosis, the answer to the following three questions should be ‘yes’: 1. Is this the worst pain you have experienced? 2. Does your pain last less than 4 h? 3. Do one or more of the following symptoms or signs occur during the headache: unilateral red eye, unilateral lacrimation, unilateral rhinorrhea, or unilateral nasal congestion? [33] Therefore, introducing the Erwin test to FM and EM physicians could help facilitate an earlier diagnosis of patients with cluster headaches.

When the questions evaluating attack treatment were examined, both groups were highly aware that oxygen should be given, while there was insufficient knowledge about medical attack treatment. The most common response in both groups was that paracetamol and NSAIDs were effective in the treatment of attacks. However, triptans are one of the most effective acute treatment options for CHs. Their subcutaneous and intranasal forms are especially preferred, because oral triptans have a slower onset of action [34,35]. Sumatriptan 6 mg s/c, zolmitriptan 5 mL nasal spray, and sumatriptan 20 mg nasal spray are the most effective acute treatments. It is not recommended to use these treatments more than twice a day [36]. Since there are no intranasal or subcutaneous forms of triptans in our country, oral triptans are preferred in the treatment of attacks. Analgesics, including opioids, have little or no place in the management of cluster headaches [36]. Dihydroergotamine has been approved by the Food and Drug Administration for acute CH treatment, although supporting evidence remains limited [37]. There is insufficient evidence to support the use of intranasal lidocaine in the acute management of CHs due to higher rates of adverse events [38]. Although subcutaneous octreotide was found to be more effective than placebo in the acute treatment of cluster headaches, it is not on the list of first-choice acute treatments [39]. Oxygen treatment is a highly effective acute treatment that can be given at home or in the emergency room at a flow of 100% oxygen at a rate of 7–15 L/min with a non-rebreather mask [40,41,42]. In our study, the EM group stated that they administered oxygen therapy with a face mask to every patient suspected of having a CH.

Transitional therapies may be used to achieve a more rapid response at the beginning of treatment. Prednisolone 75–100 mg once daily can be used for 5 days and then tapered off over 1–3 weeks [43]. Greater occipital nerve (GON) block can be used as an alternative to prednisone as a bridge treatment during the attack period [44]. It can be especially preferred in patients with CH attacks who present to the emergency department because it has few side effects, is easy to apply, and is a well-tolerated treatment [45]. When a question about the participants’ experience with GON block during the attack period was asked in the EM group, no physician stated that they had applied it. GON block can be used in the treatment of patients after training is provided to physicians in both groups, especially the EM group. Preventive treatment should be started as soon as possible after the onset of a CH attack. Drug combinations can be tried, but the physician must be careful about toxicity. In episodic CHs, preventive treatment should be discontinued by tapering 2 weeks after attack remission. In chronic CHs, treatment may need to be continued for a long time, depending on the course of the attacks. Verapamil, lithium, divalproex sodium, gabapentin, and topiramate are used for the preventive treatment of CHs [46]. Verapamil 240–960 mg/day can be used in the first-line preventive treatment of CHs, but ECG monitoring is recommended [47,48]. Lithium carbonate 600–1600 mg daily is another option for preventive therapy and serum levels must be regularly monitored [49,50]. Topiramate 50–100 mg twice daily has less evidence of efficacy, but no monitoring is required [51].

When an increase in CGRP blood levels was detected in the external jugular vein during CH attacks, it was thought that CGRP antagonists could be used in treatment, and clinical studies were conducted with different CGRP monoclonal antibodies [52,53]. Studies on Galcanezumab were terminated due to low participant numbers, and studies on Fremanezumab were terminated due to futile interim analysis results [52,54]. Studies testing the efficacy of eptinezumab in episodic CHs and erenumab in chronic CHs are ongoing [52]. Galcanezumab, licensed by the FDA but not by the European Medicines Agency for episodic CH, has studies supporting its use at doses of 240–360 mg/month, especially in refractory cases [55,56,57,58]

The EM group reported that 92.9% of the patients were evaluated in the green zone of the emergency department; therefore, the average time to start treatment was 34 min. In CHs, which are characterized as a suicidal headache, last 15–180 min, and are a very severe headache, reducing these times to more reasonable levels can be achievable through more comprehensive education of EM physicians about CHs.

Anamnesis and physical examination are necessary for differential diagnosis with other paroxysmal neurological events. Neuroimaging is also performed to exclude secondary causes in patients with atypical clinical findings [1]. However, since vascular conditions such as artery dissection and tumors may mimic a CH with their unilateral findings, neuroimaging should be performed in every patient, even if the patient presents with a typical episodic CH [33,59,60]. The EM group has a higher chance of being the patient’s first point of call during a CH attack, and the probability of rapid neuroimaging is higher. In our study, the EM group did not recommend neuroimaging in typical patients compared to the FM group, indicating that there is a lack of information on this subject and that continuous medical education is needed.

The FM group believed they could easily recognize CH patients, while the EM group was less confident about this. Although the FM group had a higher rate of confident diagnosis, both groups were wary of making a misdiagnosis. The results of this study show that physicians’ concerns about misdiagnosis are valid. Considering that even with the correct diagnosis, 25% of patients lose their jobs and 8% are unemployed due to the burden of disease, the misdiagnosis of CH leads to more dramatic consequences [30]. CHs affect the mental state of patients as well as their working lives, increasing the risk of depression and suicide [26].

All participants, more so in the EM group, thought that they could correctly direct suspected CH patients and that patients should be evaluated by a neurologist. According to a study by Voiticovschi-Iosob et al., the average time between patients being evaluated at a specialist headache center and their first attack is 4 years, but this period can be more than 10 years for 25% of patients [28]. When attacks in CHs show characteristics outside the expected course, delays in diagnosis occur more frequently. These characteristics include the occurrence of migraine-like clinical features during the attack, prolongation of attack duration exceeding 180 min, and the attacks being more frequent, especially at night [61]. While the FM group found their level of knowledge about CHs to be insufficient, the EM group stated that they were not sure of this. All participants thought that having more information would make them more confident about CHs. Our findings indicate that post-graduate education and guidelines on CHs should be better disseminated.

As in all survey studies, the most important limitation is that participants could not ask questions about items that they did not understand or about which they had a different opinion, since the survey was not conducted face to face. For this reason, first, we conducted a pilot study and tried to prepare all the questions in an easily understandable way. The lack of tests to check the internal consistency and reliability of the survey over time is an important limitation of this study.

Since participants were recruited via social media, this method may have introduced selection bias, potentially limiting the generalizability of the findings. In our study, it was seen that the participants were young, the majority were residents, and they mostly worked in training and research hospitals in both groups. The fact that some physicians did not use social media caused a decrease in the number of participants.

A strength of our study is that it evaluated the two groups with the highest probability of being the first to be contacted by CH patients. With a higher number of participants, the knowledge and opinions of EM and FM physicians about CHs could be evaluated more accurately. This study revealed the areas (such as diagnostic criteria, treatment, referral to a specialist) in which physicians felt that their knowledge about CH was inadequate. The lack of education and experience in this area should be eliminated.

## 5. Conclusions

In our study, for the first time, EM and FM physicians’ knowledge about the diagnosis and treatment of CHs, as well as their thoughts on their qualifications to treat CHs, were evaluated. It was observed that the participants had sufficient knowledge about the diagnostic criteria but had deficiencies in treatment. This study can be implemented with a higher number of participants and including different branches of medicine, such as otolaryngology, to which CH patients first present. Thus, the reasons for delayed diagnosis and misdiagnosis, which are the most basic problems in CHs, can be better understood. The early diagnosis and correct treatment of CHs, which are defined as a suicidal headache and affect patients psychologically and socioeconomically, are related to the knowledge and experience level of physicians. To avoid misdiagnosis, and for earlier and more effective treatment, EM and FM physicians, who are CH patients’ first point of call, need more medical education-based strategies related to CHs.

## Figures and Tables

**Table 1:** Comparison of family medicine and emergency medicine groups regarding the diagnosis and treatment of cluster headaches.

Statement	Answer	FM Group n: 120 (%)	EM Group n: 98 (%)	Statistical Significance
CHs are a primary headache disorder	Yes	116 (96.7)	93 (94.9)	NS
	No	4 (3.3)	5 (4.1)	
CHs are a subtype or variant of migraine	Yes	11 (9.2)	6 (6.1)	NS
	No	109 (90.8)	92 (93.9)	
Localization of CH	Unilateral	87 (72.5)	71 (72.4)	NS
	Bilateral	33 (27.5)	27 (27.6)	
CH attacks usually last	1–600 s	14 (11.7)	19 (19.4)	NS
	15–180 min	66 (55.0)	51 (52)	
	4–72 h	40 (33.3)	28 (28.6)	
Severity of headache	Moderate	39 (32.5)	31 (31.6)	NS
	Severe	81 (67.5)	67 (68.4)	
Gender predominance	Male	61 (50.8)	56 (57.1)	NS
	Female	59 (49.2)	42 (42.9)	
‘CH episodes usually occur at the same time of the year.’	True	77 (64.2)	65 (66.3)	NS
	False	10 (8.3)	5 (5.1)	
	Not sure	33 (27.5)	28 (28.6)	
‘For the diagnosis of CH, autonomic findings must always be present, and these findings should be ipsilateral and simultaneous with the pain.’	True	53 (44.2)	35 (35.7)	0.033 *
	False	49 (40.8)	34 (34.7)	
	Not sure	18 (15.0)	29 (29.6)	
‘The most common autonomic findings accompanying CH are lacrimation and conjunctival hyperemia.’	True	88 (73.3)	72 (73.5)	NS
	False	8 (6.7)	5 (5.1)	
	Not sure	24 (20.0)	21 (21.4)	
‘Even if it presents as typical episodic CH, neuroimaging is recommended for every patient with CH.’	True	61 (50.8)	29 (29.6)	0.002 *
	False	35 (29.2)	50 (51.0)	
	Not sure	24 (20.0)	19 (19.4)	
‘Paracetamol and nonsteroidal anti-inflammatory drugs are effective in CH.’	True	56 (46.7)	54 (55.1)	NS
	False	54 (45.0)	31 (31.6)	
	Not sure	10 (8.3)	13 (13.3)	
‘During a CH attack, 100% oxygen therapy is administered via a face mask for 15–20 min, at a flow rate of 7–12 L per minute.’	True	83 (69.2)	70 (45.8)	NS
	False	8 (6.7)	7 (7.1)	
	Not sure	29 (24.2)	21 (21.4)	
‘Smoking and alcohol are the most important triggers of CH.’	True	68 (56.7)	53 (54.1)	NS
	False	16 (13.3)	10 (10.2)	
	Not sure	36 (30.0)	35 (35.7)	

CH: cluster headache; EM: emergency medicine; FM: family medicine; NS: not significant. *: Pearson’s chi-square.

**Table 2:** Comparative approaches of family medicine and emergency medicine groups regarding their thoughts about their qualifications to treat cluster headaches.

Statement	Answer	FM Group n: 120 (%)	EM Group n: 98 (%)	Statistical Significance
I can easily recognize CH patients in my daily practice.	Agree	60 (50.0%)	40 (40.8%)	0.029 *
	Disagree	16 (13.3%)	6 (6.1%)	
	Not sure	44 (36.7%)	52 (53.1%)	
I can inform a patient with a suspected CH diagnosis about the necessary steps to take.	Agree	75 (62.5%)	63 (64.3%)	NS
	Disagree	11 (9.2%)	7 (7.1%)	
	Not sure	34 (28.3%)	28 (28.6%)	
A patient with CH must be referred to Neurology.	Agree	91 (75.8%)	89 (90.8%)	0.008 *
	Disagree	10 (8.3%)	1 (1.0%)	
	Not sure	19 (15.8%)	8 (8.2%)	
When encountering a patient with CH, I worry about possibly overlooking another diagnosis.	Agree	93 (77.5%)	61 (62.2%)	0.029 *
	Disagree	5 (4.2%)	11 (11.2%)	
	Not sure	22 (18.3%)	26 (26.5%)	
I am well-versed in acute attack treatments for cluster headache patients and can guide them appropriately.	Agree	51 (42.5%)	50 (51.0%)	0.003 *
	Disagree	29 (24.2%)	7 (7.1%)	
	Not sure	40 (33.3%)	41 (41.8%)	
I have adequate knowledge about CH.	Agree	15 (12.5%)	18 (18.4%)	0.006 *
	Disagree	60 (50.0%)	28 (28.6%)	
	Not sure	45 (37.5%)	52 (53.1%)	
It would make me feel more confident to have a better understanding of CH.	Agree	107 (89.2%)	83 (84.7%)	NS
	Disagree	2 (1.7%)	6 (6.1%)	
	Not sure	11 (9.2%)	9 (9.2%)	

EM: emergency medicine; FM: family medicine; NS: not significant. *: Pearson’s chi-square.

## Data Availability

The data analyzed in this research can be provided by the corresponding author upon reasonable request.

## Supplementary Materials

The following supporting information can be downloaded at https://www.mdpi.com/article/10.3390/medicina61030437/s1, The supplementary files contain survey questions for family medicine and emergency medicine physicians. Supplementary S1: Understanding of cluster headaches among family medicine doctors. Supplementary S2: Understanding of cluster headaches among emergency medicine physicians.

## References

[1] San-Juan D., Velez-Jimenez K., Hoffmann J., Martínez-Mayorga A.P., Melo-Carrillo A., Rodríguez-Leyva I., García S., Collado-Ortiz M.Á., Chiquete E., Gudiño-Castelazo M. (2024). Cluster headache: An update on clinical features, epidemiology, pathophysiology, diagnosis, and treatment. Front. Pain Res..

[2] Allena M., De Icco R., Sances G., Ahmad L., Putortì A., Pucci E., Greco R., Tassorelli C. (2019). Gender differences in the clinical presentation of cluster headache: A role for sexual hormones?. Front. Neurol..

[3] Ko C.-A., Lin G.-Y., Ting C.-H., Sung Y.-F., Lee J.-T., Tsai C.-K., Lin Y.-K., Ho T.-H., Yang F.-C. (2021). Clinical features of cluster headache: A hospital-based study in taiwan. Front. Neurol..

[4] Russell M.B. (2004). Epidemiology and genetics of cluster headache. Lancet Neurol..

[5] Fischera M., Marziniak M., Gralow I., Evers S. (2008). The incidence and prevalence of cluster headache: A meta-analysis of population-based studies. Cephalalgia.

[6] Manzoni G.C., Camarda C., Genovese A., Quintana S., Rausa F., Taga A., Torelli P. (2019). Cluster headache in relation to different age groups. Neurol. Sci..

[7] Waung M.W., Taylor A., Qualmann K.J., Burish M.J. (2020). Family history of cluster headache: A systematic review. JAMA Neurol..

[8] Malu O.O., Bailey J., Hawks M.K. (2022). Cluster headache: Rapid evidence review. Am. Fam. Physician.

[9] Headache Classification Committee of the International Headache Society (IHS) (2018). The international classification of headache disorders, 3rd edition. Cephalalgia.

[10] D’Andrea G., Gucciardi A., Perini F., Leon A. (2019). Pathogenesis of cluster headache: From episodic to chronic form, the role of neurotransmitters and neuromodulators. Headache J. Head Face Pain.

[11] Pilati L., Torrente A., Alonge P., Vassallo L., Maccora S., Gagliardo A., Pignolo A., Iacono S., Ferlisi S., Di Stefano V. (2023). Sleep and chronobiology as a key to understand cluster headache. Neurol. Int..

[12] Dias B.d.F., Robinson C.L., Villar-Martinez M.D., Ashina S., Goadsby P.J. (2024). Current and Novel Therapies for Cluster Headache: A Narrative Review. Pain Ther..

[13] Goadsby P.J., Holland P.R., Martins-Oliveira M., Hoffmann J., Schankin C., Akerman S. (2017). Pathophysiology of migraine: A disorder of sensory processing. Physiol. Rev..

[14] Bahra A., May A., Goadsby P.J. (2002). Cluster headache. Neurology.

[15] Pringsheim T. (2002). Cluster headache: Evidence for a disorder of circadian rhythm and hypothalamic function. Can. J. Neurol. Sci..

[16] Brandt R.B., Doesborg P.G.G., Haan J., Ferrari M.D., Fronczek R. (2020). Pharmacotherapy for cluster headache. CNS Drugs.

[17] Ji Lee M., Cho S.J., Wook Park J., Kyung Chu M., Moon H.S., Chung P.W., Myun Chung J., Sohn J.H., Kim B.K., Kim B.S. (2019). Increased suicidality in patients with cluster headache. Cephalalgia.

[18] Choi Y.-J., Kim B.-K., Chung P.-W., Lee M.J., Park J.-W., Chu M.K., Ahn J.-Y., Song T.-J., Sohn J.-H., Oh K. (2018). Impact of cluster headache on employment status and job burden: A prospective cross-sectional multicenter study. J. Headache Pain.

[19] Pohl H., Gantenbein A.R., Sandor P.S., Schoenen J., Andrée C. (2022). Cluster headache and the comprehension paradox. SN Compr. Clin. Med..

[20] Donnet A., Lanteri-Minet M., Guegan-Massardier E., Mick G., Fabre N., Geraud G., Lucas C., Navez M., Valade D., Société Française d’Etude des Migraines et Céphalées (SFEMC) (2007). Chronic cluster headache: A French clinical descriptive study. J. Neurol. Neurosurg. Psychiatry.

[21] Lund N.L., Snoer A.H., Jensen R.H. (2019). The influence of lifestyle and gender on cluster headache. Curr. Opin. Neurol..

[22] Lund N., Petersen A., Snoer A., Jensen R.H., Barloese M. (2018). Cluster headache is associated with unhealthy lifestyle and lifestyle-related comorbid diseases: Results from the Danish Cluster Headache Survey. Cephalalgia.

[23] Barloese M.C.J., Jennum P.J., Lund N.T., Jensen R.H. (2015). Sleep in cluster headache—Beyond a temporal rapid eye movement rela-tionship?. Eur. J. Neurol..

[24] Barloese M. (2021). Current understanding of the chronobiology of cluster headache and the role of sleep in its management. Nat. Sci. Sleep.

[25] Bahra A., Goadsby P.J. (2004). Diagnostic delays and mis-management in cluster headache. Acta Neurol. Scand..

[26] Buture A., Ahmed F., Mehta Y., Paemeleire K., Goadsby P.J., Dikomitis L. (2020). Perceptions, experiences, and understandings of cluster headache among GPs and neurologists: A qualitative study. Br. J. Gen. Pract..

[27] Li Y.-M., Ren L.-N., Xu X.-F., Dai Y.-L., Jin C.-Q., Yang R.-R. (2024). Cluster headache: Understandings of current knowledge and directions for whole process management. Front. Neurol..

[28] Voiticovschi-Iosob C., Allena M., De Cillis I., Nappi G., Sjaastad O., Antonaci F. (2014). Diagnostic and therapeutic errors in cluster headache: A hospital-based study. J. Headache Pain.

[29] Van Alboom E., Louis P., Van Zandijcke M., Crevits L., Vakaet A., Paemeleire K. (2009). Diagnostic and therapeutic trajectory of cluster headache patients in Flanders. Acta Neurol. Belg..

[30] May A., Schwedt T.J., Magis D., Pozo-Rosich P., Evers S., Wang S.-J. (2018). Cluster headache. Nat. Rev. Dis. Prim..

[31] Vollesen A.L.H., Snoer A., Beske R.P., Guo S., Hoffmann J., Jensen R.H., Ashina M. (2018). Effect of infusion of calcitonin gene-related peptide on cluster headache attacks: A randomized clinical trial. JAMA Neurol..

[32] Long R.-J., Zhu Y.-S., Wang A.-P. (2021). Cluster headache due to structural lesions: A systematic review of published cases. World J. Clin. Cases.

[33] Parakramaweera R., Evans R.W., I Schor L., Pearson S.M., Martinez R., Cammarata J.S., Amin A.J., Yoo S.-H., Zhang W., Yan Y. (2021). A brief diagnostic screen for cluster headache: Creation and initial validation of the Erwin Test for Cluster Headache. Cephalalgia.

[34] Wei D.Y., Goadsby P.J. (2021). Cluster headache pathophysiology—Insights from current and emerging treatments. Nat. Rev. Neurol..

[35] Robbins M.S., Starling A.J., Pringsheim T.M., Becker W.J., Schwedt T.J. (2016). Treatment of Cluster Headache: The American Headache Society Evidence-Based Guidelines. Headache.

[36] Steiner T.J., Jensen R., Katsarava Z., Linde M., MacGregor E.A., Osipova V., Paemeleire K., Olesen J., Peters M., Martelletti P. (2019). Aids to management of headache disorders in primary care (2nd edition): On behalf of the European Headache Federation and Lifting The Burden: The Global Campaign against Headache. J. Headache Pain.

[37] Nagy A.J., Gandhi S., Bhola R., Goadsby P.J. (2011). Intravenous dihydroergotamine for inpatient management of refractory primary headaches. Neurology.

[38] Dagenais R., Zed P.J. (2018). Intranasal Lidocaine for Acute Management of Primary Headaches: A Systematic Review. Pharmacotherapy.

[39] Matharu M.S., Levy M.J., Meeran K., Goadsby P.J. (2004). Subcutaneous octreotide in cluster headache: Randomized placebo-controlled double-blind crossover study. Ann. Neurol..

[40] Evers S., Rapoport A. (2016). The use of oxygen in cluster headache treatment worldwide—A survey of the International Headache Society (IHS). Cephalalgia.

[41] Dirkx T.H.T., Haane D.Y.P., Koehler P.J. (2018). Oxygen treatment for cluster headache attacks at different flow rates: A double-blind, randomized, crossover study. J. Headache Pain.

[42] Petersen A.S., Lund N., Jensen R.H., Barloese M. (2020). Real-life treatment of cluster headache in a tertiary headache center—Results from the Danish Cluster Headache Survey. Cephalalgia.

[43] Lund N.L.T., Petersen A.S., Fronczek R., Tfelt-Hansen J., Belin A.C., Meisingset T., Tronvik E., Steinberg A., Gaul C., Jensen R.H. (2023). Current treatment options for cluster headache: Limitations and the unmet need for better and specific treatments—A consensus article. J. Headache Pain.

[44] Hasırcı Bayır B.R., Gürsoy G., Sayman C., Yüksel G.A., Çetinkaya Y. (2022). Greater occipital nerve block is an effective treat-ment method for primary headaches?. Agri.

[45] Gordon A., Roe T., Villar-Martínez M.D., Moreno-Ajona D., Goadsby P.J., Hoffmann J. (2023). Effectiveness and safety profile of greater occipital nerve blockade in cluster headache: A systematic review. J. Neurol. Neurosurg. Psychiatry.

[46] Orvin C.A., Zaheri S.C., Perilloux D.M., Field E., Ahmadzadeh S., Shekoohi S., Kaye A.D. (2024). Divalproex, Valproate, & Developing Treatment Options for Cluster Headache Prophylaxis: Clinical Practice Considerations. SN Compr. Clin. Med..

[47] Leone M., D’amico D., Frediani F., Moschiano F., Grazzi L., Attanasio A., Bussone G. (2000). Verapamil in the prophylaxis of episodic cluster headache: A double-blind study versus placebo. Neurology.

[48] Blau J.N., Engel H.O. (2004). Individualizing Treatment with Verapamil for Cluster Headache Patients. Headache.

[49] Peng K.-P., Burish M.J. (2023). Management of cluster headache: Treatments and their mechanisms. Cephalalgia.

[50] Nikolova V.L., Pattanaseri K., Hidalgo-Mazzei D., Taylor D., Young A.H. (2018). Is lithium monitoring NICE? Lithium monitoring in a UK secondary care setting. J. Psychopharmacol..

[51] Leone M., Dodick D., Rigamonti A., D’Amico D., Grazzi L., Mea E., Bussone G. (2003). Topiramate in Cluster Headache Prophylaxis: An Open Trial. Cephalalgia.

[52] Steiner T.J., Jensen R., Katsarava Z., Linde M., MacGregor E.A., Osipova V., Paemeleire K., Olesen J., Peters M., Martelletti P. (2023). Hellenic Headache Society Recommendations for the Use of Monoclonal Antibodies Targeting the Calcitonin Gene-Related Peptide Pathway for the Prevention of Migraine and Cluster Headache—2023 Update. SN Compr. Clin. Med..

[53] Pérez R.L., Millán-Vázquez M., González-Oria C. (2024). Efficacy and safety of galcanezumab as chronic cluster headache preventive treatment under real world conditions: Observational prospective study. Cephalalgia.

[54] Goadsby P.J., Dodick D.W., Leone M., Bardos J.N., Oakes T.M., Millen B.A., Zhou C., Dowsett S.A., Aurora S.K., Ahn A.H. (2019). Trial of galcanezumab in prevention of episodic cluster headache. N. Engl. J. Med..

[55] Argyriou A.A., Vikelis M., Mantovani E., Litsardopoulos P., Tamburin S. (2020). Recently available and emerging therapeutic strategies for the acute and prophylactic management of cluster headache: A systematic review and expert opinion. Expert Rev. Neurother..

[56] Membrilla J.A., Torres-Ferrus M., Alpuente A., Caronna E., Pozo-Rosich P. (2022). Efficacy and safety of galcanezumab as a treatment of refractory episodic and chronic cluster headache: Case series and narrative review. Headache J. Head Face Pain.

[57] Riesenberg R., Gaul C., Stroud C.E., Dong Y., Bangs M.E., Wenzel R., Martinez J.M., Oakes T.M. (2022). Long-term open-label safety study of galcanezumab in patients with episodic or chronic cluster headache. Cephalalgia.

[58] Mo H., Kim B.K., Moon H.S., Cho S.J. (2022). Real-world experience with 240 mg of galcanezumab for the preventive treatment of cluster headache. J. Headache Pain.

[59] Mitsikostas D.D., Ashina M., Craven A., Diener H.C., Goadsby P.J., Ferrari M.D., Lampl C., Paemeleire K., Pascual J., Siva A. (2015). European headache federation consensus on technical investigation for primary headache disorders. J. Headache Pain.

[60] Expert Panel on Neurologic Imaging (2019). Whitehead, M.T.; Cardenas, A.M.; Corey, A.S.; Policeni, B.; Burns, J.; Chakraborty, S.; Crowley, R.W.; Jabbour, P.; Ledbetter, L.N.; et al. ACR Appropriateness Criteria^®^ Headache. J. Am. Coll. Radiol..

[61] Wei D.Y., Khalil M., Goadsby P.J. (2019). Managing cluster headache. Pract. Neurol..

