# Supplementary material for: Cluster Headache Management: Evaluating Diagnostic and Treatment Approaches Among Family and Emergency Medicine Physicians
Source: Medicina (Kaunas). 2025 Feb 28;61(3):437. doi: 10.3390/medicina61030437 (PMC11943940; doi:10.3390/medicina61030437)
Supplement: Supplementary file 1 [file medicina-61-00437-s001.zip › medicina-3395363-supplementary.pdf]

SUPPLEMENTARY-S1:

UNDERSTANDING OF CLUSTER HEADACHE (CH) AMONG FAMILY MEDICINE DOCTORS

1) How old are you?

2) What is your gender?
    A. Male
    B. Female

3) Which of the following do you belong to?
    A. Family Medicine resident doctor
    B. Family Medicine specialist doctor

4) How long have you been working? (in years)

5) Where do you work? (name of the facility)

6) Is CH a primary headache or a secondary headache disorder?
    A. Primary headache (In primary headache, there is no underlying etiology)
    B. Secondary headache (In secondary headache, headache is secondary to another condition like brain tumor, stroke or metabolic disorder)

7) Is CH a subtype or variant of migraine?
    A. No
    B. Yes

8) Which statement suits best regarding the localization of CH?
    A. Unilateral
    B. Bilateral

9) How long does one CH attack usually last?
    A. 1-600 seconds
    B. 15-180 minutes
    C. 4-72 hour

10) How severe is the pain of CH?
    A. Moderate
    B. Severe

11) Which gender is more commonly affected by CH?
    A. Male
    B. Female

*12-20: Please evaluate the following statements as true, false, or not sure.

12) ‘CH episodes usually occur at the same time of the year.’

A. True
B. False
C. Not sure

13) ‘For the diagnosis of CH, autonomic findings must always be present and these findings should be ipsilateral and simultaneous with the pain.’
A. True
B. False
C. Not sure

14) ‘The most common autonomic findings accompanying CH are lacrimation and conjunctival hyperemia.’
A. True
B. False
C. Not sure

15) ‘Even if it presents as typical episodic CH, neuroimaging is recommended for every patient with CH.’
A. True
B. False
C. Not sure

16) ‘During a CH attack, 100% oxygen therapy is administered via a face mask for 15-20 minutes, at a flow rate of 7-12 liters per minute.’
A. True
B. False
C. Not sure

17) ‘Paracetamol and nonsteroidal anti-inflammatory drugs are effective in CH.’
A. True
B. False
C. Not sure

18) ‘Smoking and alcohol are the most important triggers of CH.’
A. True
B. False
C. Not sure

19) Do you use the International Headache Society’s headache classification system while diagnosing the cluster headache?
A. No
B. Yes

* Evaluate the statements in the table below by selecting the option that best suits you.

	**Agree**	**Disagree**	**Not sure**
**20) I can easily recognize CH patients in my daily practice.**			

	Agree	Disagree	Not sure
**21) I believe I can inform a patient with a suspected CH diagnosis about the necessary steps to take.**			
**22) I believe that a patient with CH must be referred to Neurology.**			
**23) When encountering a patient with CH, I worry about possibly overlooking another diagnosis.**			
**24) If a CH patient is having an acute attack, I refer them to the Emergency Department.**			
**25) I believe I am well-versed in the acute attack treatments for cluster headache patients and can guide them appropriately.**			
**26) I have adequate knowledge about non-pharmacological approaches for CH patients.**			
**27) I have adequate knowledge about the prophylaxis of CH patients.**			
**28) I believe I have adequate knowledge about CH.**			
**29) It would make me feel more confident in my daily practice to have a better understanding of CH.**			

SUPPLEMENTARY-S2:

UNDERSTANDING OF CLUSTER HEADACHE (CH) AMONG EMERGENCY MEDICINE PHYSICIANS

1) How old are you?

2) What is your gender?
    A. Male
    B. Female

3) Which of the following do you belong to?
    A. Emergency Medicine resident doctor
    B. Emergency Medicine specialist doctor

4) How long have you been working? (in years)

5) Where do you work? (name of the facility)

6) Is CH a primary headache or a secondary headache disorder?
    A. Primary headache (In primary headache, there is no underlying etiology)
    B. Secondary headache (In secondary headache, headache is secondary to another condition like brain tumor, stroke or metabolic disorder)

7) Is CH a subtype or variant of migraine?
    A. No
    B. Yes

8) Which statement suits best regarding the localization of CH?
    A. Unilateral
    B. Bilateral

9) How long does one CH attack usually last?
    A. 1-600 seconds
    B. 15-180 minutes
    C. 4-72 hours

10) How severe is the pain of CH?
    A. Moderate
    B. Severe

11) Which gender is more commonly affected by CH?
    A. Male
    B. Female

*12-25 : Please evaluate the following statements as true, false, or not sure.

12) 'CH episodes usually occur at the same time of the year.'
    A. True
    B. False

C. Not sure

13) 'For the diagnosis of CH, autonomic findings must always be present and these findings should be ipsilateral and simultaneous with the pain.'

A. True
B. False
C. Not sure

14) 'The most common autonomic findings accompanying CH are lacrimation and conjunctival hyperemia.'

A. True
B. False
C. Not sure

15) 'Even if it presents as typical episodic CH, neuroimaging is recommended for every patient with CH.'

A. True
B. False
C. Not sure

16) 'During a CH attack, 100% oxygen therapy is administered via a face mask for 15-20 minutes, at a flow rate of 7-12 liters per minute.'

A. True
B. False
C. Not sure

17) Do you administer oxygen therapy with a face mask to every patient in whom you suspect CH?

A. No
B. Yes

18) 'Paracetamol and nonsteroidal anti-inflammatory drugs are effective in CH.'

A. True
B. False
C. Not sure

19) 'Triptans (5HT1B/D receptor agonists) used in migraine treatment are effective in CH.'

A. True
B. False
C. Not sure

20) 'Smoking and alcohol are the most important triggers of CH.'

A. True
B. False
C. Not sure

21) Do you use the International Headache Society's headache classification system while diagnosing headache syndromes?

A. No
B. Yes

22) Did you use the International Headache Society's diagnostic criteria for CH?

A. No
B. Yes

23) In which area of the emergency department do you monitor patients suspected of having CH?

A. Green zone
B. Red zone

24) How many minutes on average do patients suspected of having CH receive treatment after presenting to the emergency department?

25) Have you performed GON block (Greater Occipital Nerve Block) on a patient suspected of having cluster headache?

A. No
B. Yes

*Evaluate the statements in the table below by selecting the option that best suits you.

	Agree	Disagree	Not sure
26) I can easily recognize CH patients in my daily practice.			
27) I believe I can inform a patient with a suspected CH diagnosis about the necessary steps to take.			
28) I believe that a patient with CH must be referred to Neurology.			
29) When encountering a patient with CH, I worry about possibly overlooking another diagnosis.			
30) I believe I am well-versed in the acute attack treatments for cluster headache patients and can guide them appropriately.			
31) I believe I have adequate knowledge about CH.			

	Agree	Disagree	Not sure
**32) It would make me feel more confident in my daily practice to have a better understanding of CH.**			
